# Highly stable pulsed fiber laser generation modulated by chromium iodide film

**DOI:** 10.1515/nanoph-2023-0530

**Published:** 2023-11-02

**Authors:** Ning Li, Lin Du, Dongyang Liu, Jiadong Wu, Yuan He, Yi Feng, Man Wu, Lili Miao, Chujun Zhao

**Affiliations:** State Key Laboratory for Micro/Nano Optoelectronic Devices of Ministry of Education & Hunan Provincial Key Laboratory of Low-Dimensional Structural Physics and Devices. School of Physics and Electronics, Hunan University, Changsha 410082, China; School of Physics and Electronic Science, Hunan University of Science and Technology, Xiangtan 411201, China

**Keywords:** mode-locked fiber laser, mid-infrared laser, nonlinear optics, broadband saturation absorption

## Abstract

Highly stable pulsed fiber lasers are key optical components in optical communication, optical sensing, and precision micromachining systems due to the high beam quality, high peak power, and compact configurations. However, the available optical modulators in the fiber laser suffer from the operation bandwidth limitations and poor long-term physicochemical stability. Here, we have investigated the broadband nonlinear optical absorption behavior of the chromium iodide (CrI_3_) film, which exhibits broadband saturable absorption towards the mid-infrared regime and excellent long-term stability. The conventional soliton fiber laser operating at telecom wavelength has been obtained from an Er^3+^-doped fiber laser (EDFL) utilizing CrI_3_ film with a signal-to-noise ratio (SNR) of 92.4 dB and a pulse width of 492 fs. In addition, a passively Q-switched operation around 2.8 μm has also been obtained from an Er^3+^-doped ZBLAN fiber laser (EDZFL) modulated by the CrI_3_ film with a SNR of 46.8 dB and a pulse width of 766 ns. The demonstration shows that the CrI_3_ film exhibits robust broadband optical modulation, and may make inroads for developing highly stable ultrafast optoelectronic devices.

## 1 Introduction

The highly stable pulsed fiber lasers have drawn great attention for the versatile applications in optical communication, optical sensing, precision micromachining for the compact configuration, high beam quality, high peak power, and so on [1–3]. Usually, the pulsed fiber lasers can be realized via the nonlinear optical modulation behavior of the saturable absorber (SA), which exhibit intensity-dependent optical absorption. Up to now, different optical materials have been studied to show broadband optical response to modulate the fiber lasers successfully [4–9]. However, the ultrafast lasers based on SAs still face challenges, such as poor long-term stability, low damage threshold and complicated manufacturing procedures, which are essential for practical applications. Inspired by the requirement for highly stable, cost-effective pulsed fiber lasers, it is urgent to explore SAs with broadband optical response, high physicochemical stability, fast relaxation time, and strong optical nonlinearity.

Chromium iodide (CrI_3_), one of the few insulating ferromagnets, has attracted considerable attention in the field of magnetism [10–15]. The Cr^3+^ ions in CrI_3_ are in an octahedral honeycomb network of six I^−^ ions, where each I^−^ ion is bonded to two Cr^3+^ ions. The formed CrI_3_ slabs are stacked with van der Waals (vdW) gaps separating them [16]. In addition, CrI_3_ is stable both in air and in water without the contamination of CrI_2_. The unique layer-dependent magnetic property of CrI_3_ means it can serve as an excellent platform for studying truly two-dimensional ferromagnets [10]. Subsequent studies have mostly focused on how to control its magnetic nature [17–21]. Thus far, different magnetic and optoelectronic applications of CrI_3_ have been reported, such as tunneling magnetoresistance [14, 15, 22], electrostatic doping [17], helical luminescence [23], and light helicity detector [24]. Particularly, chromium trihalides have been discovered to act as Mott insulators in previous research, and their optical response is determined by charge-transfer transitions and ligand-field [25–28]. Further research has shown that the incident photon can influence the excitonic transitions in CrI_3_ [29], which is a key step in the application of high-performance optoelectronic devices. As a vdW-bonded layered magnetic material, CrI_3_ film is an optoelectronic material with the advantages of good optical absorptions, high stability, broadband optical response and ease of manufacturing, which make it a better material to be applied in the field of ultrafast laser compared with other two-dimensional materials like black phosphorus and its analogues [30–32]. However, there is still no research on the potential of CrI_3_ in the field of nonlinear optics and application in ultrafast photonics.

Here, we have prepared the CrI_3_ film and investigated the broadband linear and nonlinear absorption of CrI_3_ film, validating the capability of CrI_3_ film for broadband optical modulation. Notably, with the modulation of the CrI_3_ film, the mode-locked EDFL operating at telecom wavelength has been obtained with a pulse duration of 492 fs, fundamental repetition rate of 28.2 MHz and SNR of 92.4 dB, respectively. By applying the CrI_3_-based SA to EDZFL operating in the mid-infrared regime, the Q-switched operation has been delivered with a SNR of 46.8 dB and pulse duration of 766 ns, respectively. The broadband optical modulation and extraordinary stability illustrate the promising applications of CrI_3_ film in the broadband nonlinear optoelectronics.

## 2 Fabrication and characterization of the CrI_3_ film

**Figure 1:**
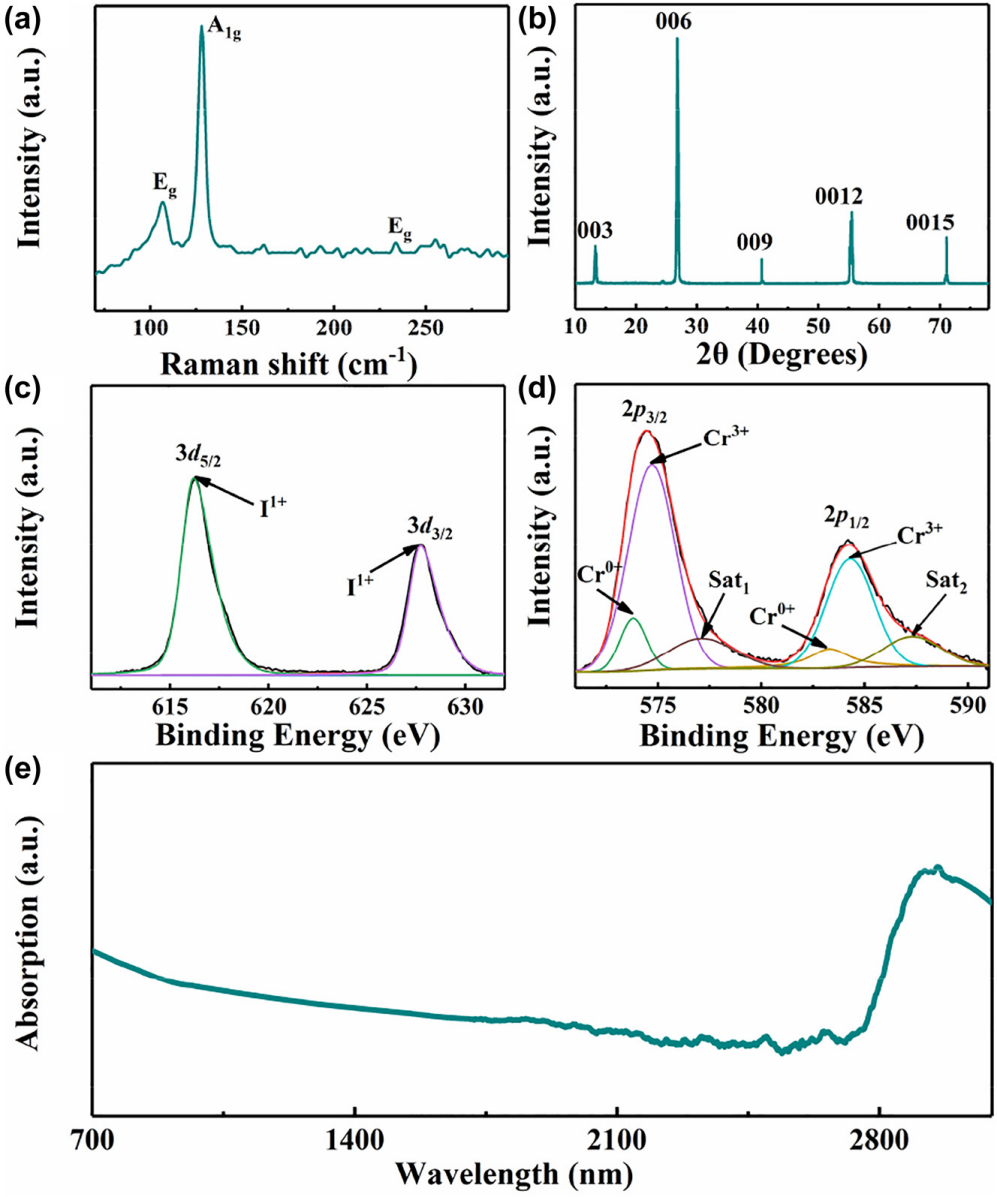
Characterization of the CrI_3_ film. (a) The Raman spectra of the CrI_3_ film. (b) XRD patterns of the CrI_3_ film. (c), (d) XPS spectra of I (3d) and Cr (2p) for the obtained CrI_3_ film, respectively. (e) The linear absorption spectrum of the CrI_3_ film.

The CrI_3_ film used in the experiment was prepared by the spin-coating method. The commercial CrI_3_ powder was dissolved in alcohol to obtain a solution and then spun into a film. The Raman spectra of the CrI_3_ film in Figure 1(a) display a series of peaks at the frequencies of 106.5, 128.4, and 231.7 cm^−1^, consistent with results in the previous work [33]. The XRD patterns presented in Figure 1(b) provide compelling evidence that the CrI_3_ film exhibits a triangular structure that is ordered within an *R* $\bar{3}$ space group. The diffraction peaks within the patterns are shown to match well with prior research [16]. To verify the element composition of the CrI_3_ film, the X-ray photoelectron spectroscopy (XPS) was adopted, whose results are depicted in Figure 1(c) and (d). After decomposing by Voigt function, the results were dealt with Shirley background correction procedure. The two peaks in Figure 1(c) locating at binding energies (BEs) of 616.2 and 627.7 eV correspond to the core levels of I^1+^_5/2_ and I^1+^_3/2_. In Figure 1(d), there are four peaks at the BEs of 573.8, 574.7, 583.3, and 584.3 eV, corresponding to the core levels of Cr^0+^_3/2_, Cr^3+^_3/2_, Cr^0+^_1/2_, and Cr^3+^_1/2_, respectively. The XPS results imply the fabricated film is mainly composed of CrI_3_ and contain a small amount of Cr metals. The linear absorption spectrum of CrI_3_ film depicted in Figure 1(e) manifests a broadband absorption, especially a significant absorption peak in the mid-infrared wavelength band, indicating that CrI_3_ film can act as an ideal optical material for broadband optical response towards mid-infrared regime.

**Figure 2:**
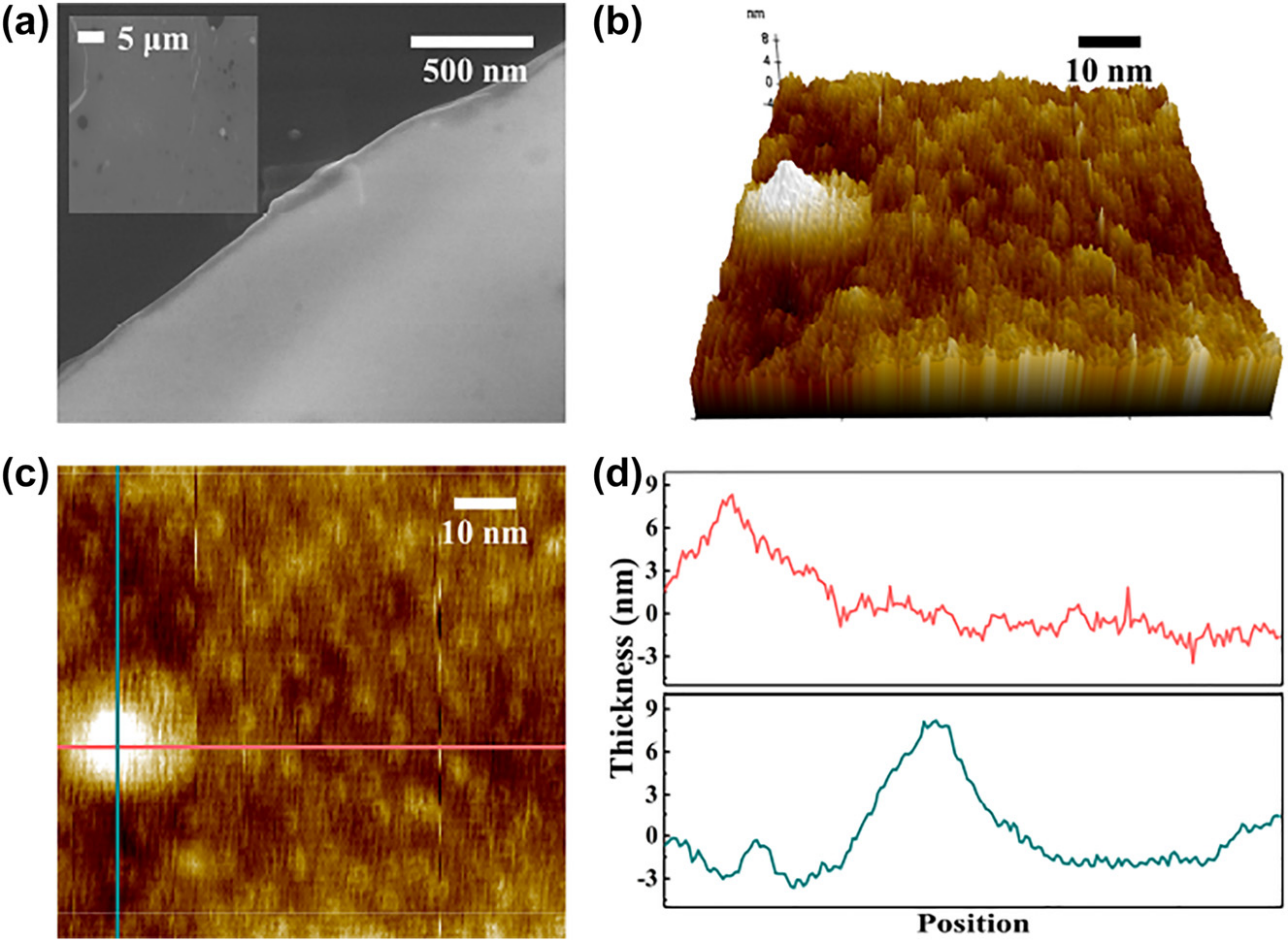
The SEM and AFM results of the CrI_3_ film. (a) SEM image of the CrI_3_ film, and the inset shows the SEM image with a large scale. (b)–(d) The AFM results of the CrI_3_ film.

The surface morphology of the CrI_3_ film was analyzed using scanning electron microscope (SEM). Figure 2(a) depicts the resulting image, revealing that the CrI_3_ film possesses a layered structure with high levels of uniformity and continuity [10]. The thickness of the CrI_3_ film has been measured to be 9 nm by using atomic force microscope (AFM), whose results are depicted in Figure 2(b)–(d).

**Figure 3:**
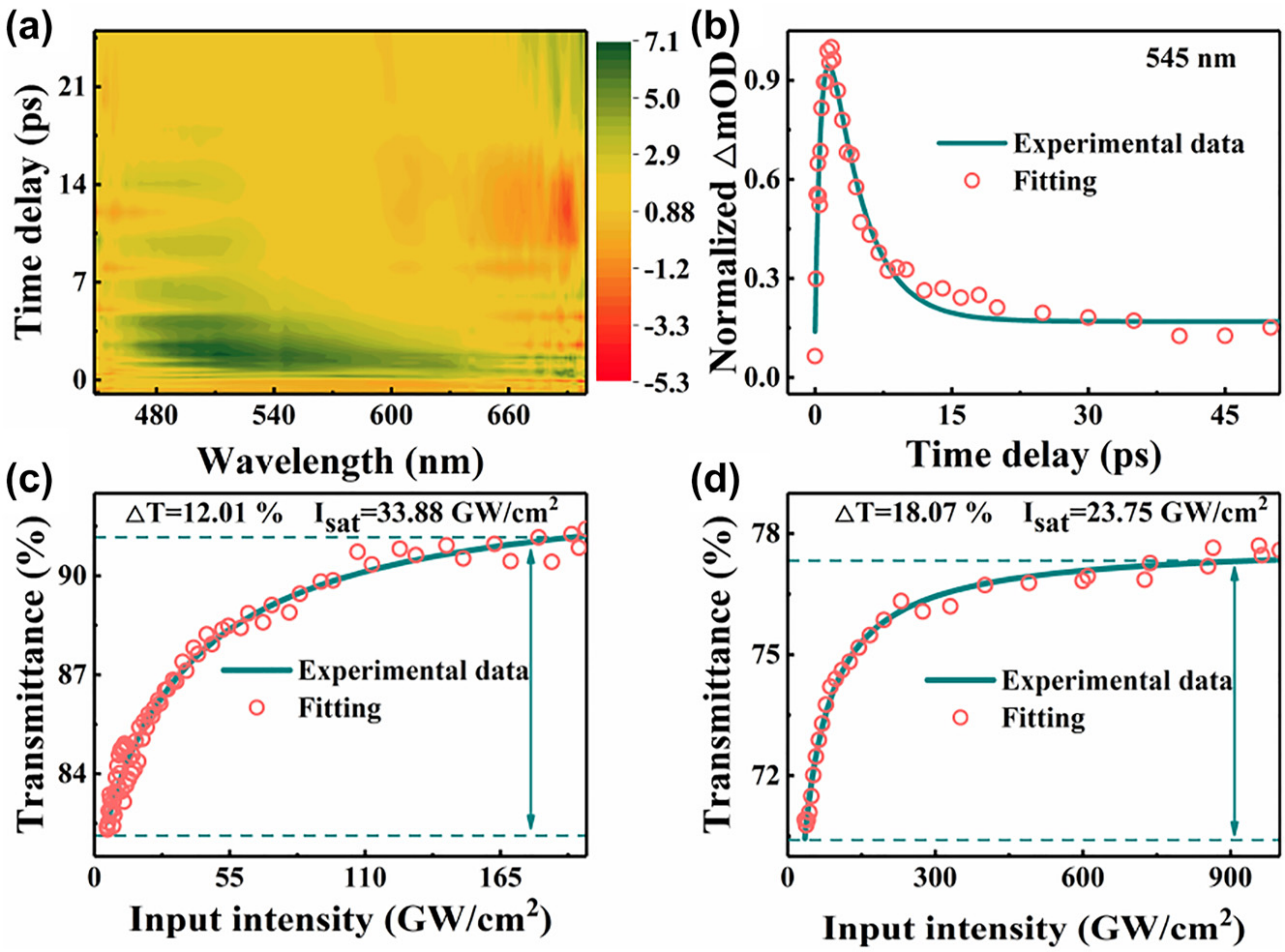
The absorption characteristics of the CrI_3_ film. (a) Time-resolved transient absorption spectroscopy of CrI_3_ film. (b) Ultrafast carrier dynamic of CrI_3_ film. The nonlinear saturable absorption behavior at the wavelength of (c) 1550 nm and (d) 2800 nm, respectively.

The transient absorption spectrum of the CrI_3_ film has been explored to investigate the ultrafast carrier dynamics of CrI_3_ film by a ultrafast laser with a center wavelength of 400 nm, repetition rate of 6 kHz, and pulse duration of 190 fs, as shown in Figure 3(a) and (b). The experimental result has been fitted by the following formula:

(1) $$y=A_{1}\exp\left(-\frac{t}{\tau_{1}}\right)+A_{2}\exp\left(-\frac{t}{\tau_{2}}\right)$$

where *τ*_1_, *τ*_2_ represent fast and slow decay time, which were extracted to 0.73 ps and 3.73 ps, respectively. The fast decay time can be attributed to the intraband carrier–carrier scattering in the CrI_3_ film, and the slow decay time is relevant to the carrier-photon scattering process. In addition, the fast relaxation time further demonstrates the potential of CrI_3_ film in ultrafast laser devices. The nonlinear optical absorption behavior of the CrI_3_ film has been measured by the Z-scan technique. By fitting the curves, the modulation depth and saturation intensity of CrI_3_ film were 12.01 % and 33.88 GW/cm^2^ at 1550 nm, 18.07 % and 23.75 GW/cm^2^ at 2800 nm, respectively, as depicted in Figure 3(c) and (d), which shows the strong broadband nonlinear optical modulation of the film. In addition, it can also be concluded from Figure 3(c) and (d) that the CrI_3_ film can withstand the incident intensity at least 165 GW/cm^2^ at 1.55 μm and 900 GW/cm^2^ at 2.8 μm, respectively.

## 3 Experimental results and discussions

### 3.1 Mode-locked operation around 1550 nm

**Figure 4:**
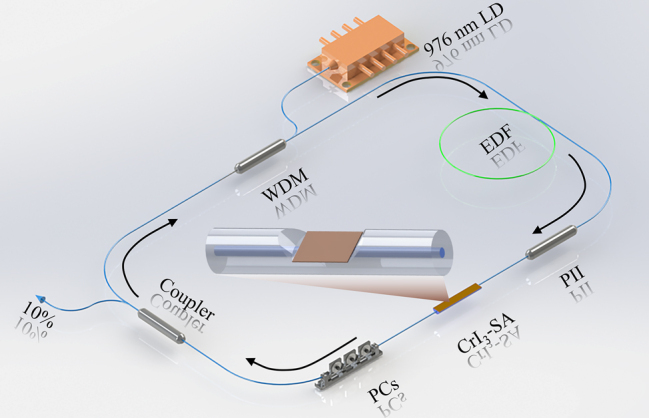
The schematic diagram of the experimental setup of EDFL. CrI_3_ film-SA: Chromium Iodinates saturable absorber.

Figure 4 displays the schematic diagrammatic illustration of the Er^3+^-doped mode-locked fiber laser at 1550 nm, which consists of a pump laser, a wavelength division multiplexer (WDM), an Er^3+^-doped fiber (EDF, LIEKKI 80-8/125) serving as the gain fiber, a polarization-independent isolator (PII) that enables the unidirectional transmission of the laser within the cavity, a side polished fiber coated with CrI_3_ film as SA, polarization controllers (PCs) to optimize the birefringence within cavity and a 90:10 optical coupler where the 10 % port was utilized as the laser output port. The fiber ring cavity has a length of 7.3 m with 1.0 m being composed of EDF and 6.3 m composed of SMF-28 single-mode fiber. The insertion loss of the CrI_3_ film SA has been evaluated to be 2.2 dB in this experiment. The characteristics of the output have been detected by an autocorrelator (APE Pulsecheck), a digital oscilloscope (Agilent DSO9404A), a radio frequency (RF) spectrum analyzer (KEYSIGHT CXA Signal Analyzer N9000B) and an optical spectrum analyzer (YOKOGAWA AQ6370D).

**Figure 5:**
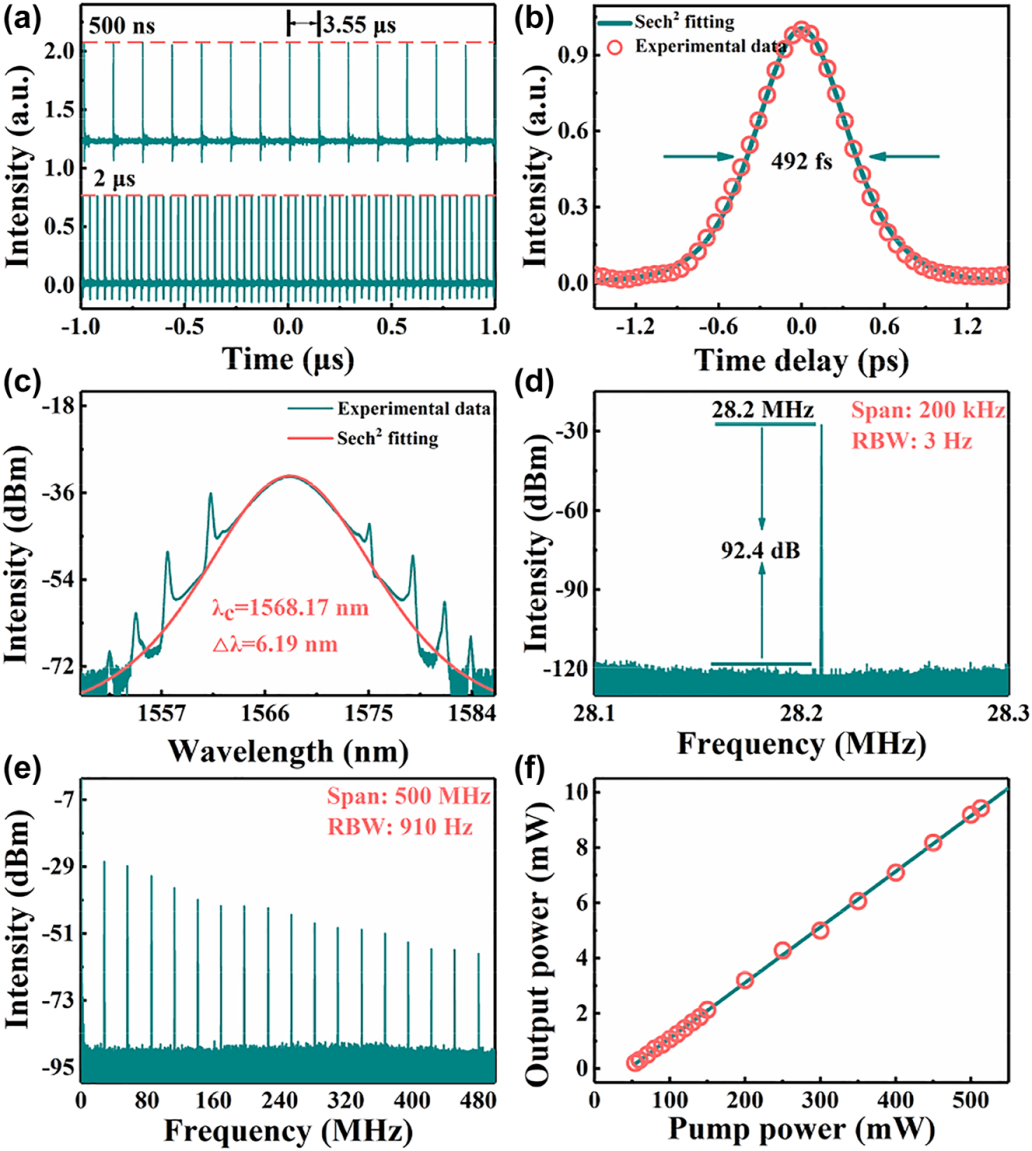
The experimental results of the EDFL based on CrI_3_ film SA. (a) The temporal pulse train with different time spans, bottom: 2 μs, top: 500 ns. (b) The autocorrelation traces. (c) The optical spectrum. (d) (e) The RF spectrum with different spans and RBWs. (f) Variation of output power with pump power.

The self-starting mode-locking operation can be obtained when the pump power exceeds the threshold of 54 mW, and the stable mode-locked operation appears at the pump power of 90 mW by carefully adjusting the state of the PCs. The results obtained at this state are depicted in Figure 5. The uniform output of the pulse train shown in Figure 5(a) manifests the good stability of the fiber laser, and the pulse interval of 3.55 μs can correspond well with the fundamental repetition rate of 28.2 MHz. The autocorrelation trace of the output in Figure 5(b) reveals that the pulse width is 492 fs after fitting with the Sech^2^ function. Figure 5(c) shows the output spectrum and the processed spectrum using the Sech^2^ function, indicating a 3-dB width of 6.19 nm and a center wavelength of 1568.17 nm. The presence of Kelly sidebands as seen in Figure 5(c) is a typical characteristic of a fiber laser operating in conventional soliton regime. The measured radio-frequency (RF) spectrums shown in Figure 5(d) and (e) with different resolution bandwidths (RBWs) and spans manifest the extraordinary stability of the fiber laser. The high SNR of 92.4 dB of the ultrafast pulse at a fundamental repetition rate of 28.2 MHz was demonstrated successfully, which indicates that the cavity is capable of producing a very stable and robust pulse for practical applications. The experiments also show that there is an almost constant slope between the pump power and output power, which is depicted in Figure 5(f). Even at power about 500 mW, there was no significant degradation of output power observed, suggesting that the cavity can handle high power inputs without compromising its performance.

**Figure 6:**
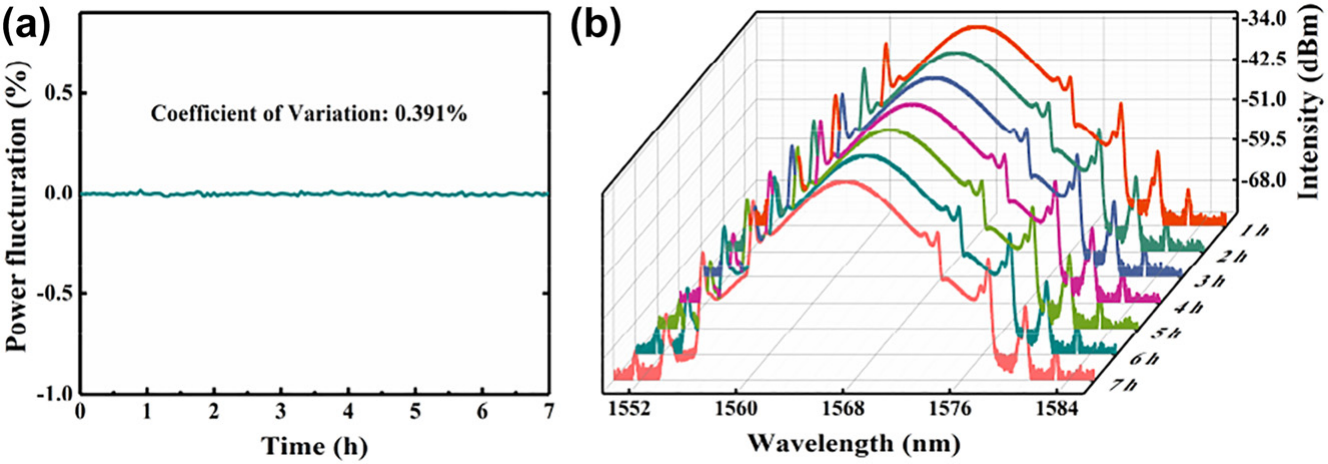
The operation stability of the EDFL. (a) The power fluctuation of the output power and (b) the long-term stability of the output spectra.

Moreover, a long-term operation ability has been tested to investigate the stability of the EDFL based on CrI_3_ film-SA, as shown in Figure 6. The spectra and the output power have been monitored for seven consecutive hours. The coefficient of variation calculated in Figure 6(a) is 0.391 %, and the spectra sampled per hour shown in Figure 6(b) maintain good consistency in shape, manifesting the reliable effectiveness and durability of the CrI_3_ film-SA. The CrI_3_ film SA device shows good environmental stability. After several days in the ambient environment, the high stability mode-locked output can also be obtained.

### 3.2 Q-switched operation around 3 μm

**Figure 7:**
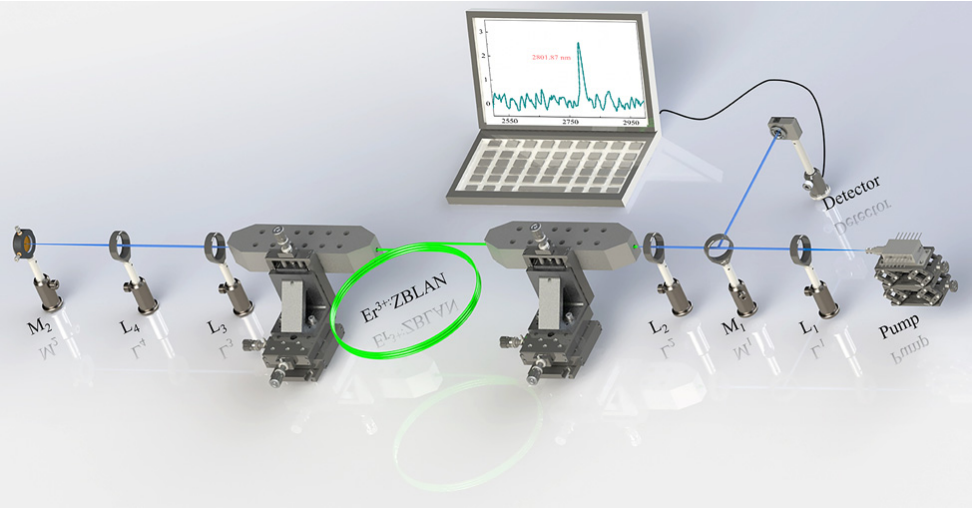
The schematic diagram of the EDZFL.

Encouraged by the absorption behavior of the CrI_3_ film near 2.8 μm, we have further elaborately designed an EDZFL to investigate the usage of CrI_3_ film as SA in the mid-infrared regime. Figure 7 displays the diagrammatic illustration of the Q-switched EDZFL, in which the pump source is a diode laser with a wavelength of 975 nm, NA of 0.22, core diameter of 105 μm and maximum output power of 40 W. A collimation-focusing system consisting of plano-convex lenses *L*_1_ (N-BK7, *f* = 25.4 mm) and *L*_2_ (CaF_2_, *f* = 25.4 mm) was adopted to guide the laser from the pump into the 3.8 m-double-cladding fluoride fiber with inner clad of 240 × 260 μm (core diameter: 15 μm, NA: 0.12, Le Verre Fluoré). *L*_3_ (uncoated CaF_2_, *f* = 20 mm) and L_4_ (uncoated CaF_2_, *f* = 25.4 mm) constituted another collimation-focusing system to focus the laser onto the CrI_3_ film-SA which was spin-coated on the M_2_ (PF 10-03-M01, Thorlabs). The pulsed laser can be extracted through a dichroic mirror (M_1_) with high reflectivity at 2.8 μm and high transmittance at 975 nm. The insertion loss of the CrI_3_ film SA has been evaluated to be 3.4 dB at 2.8 μm wavelength.

At the pump power of approximately 500 mW, the Q-switched operation can be obtained successfully. When the pump power exceeded 1.5 W, a stable Q-switched pulse sequence occurred, which can maintain until the pump power increased to 2.7 W. For the mid-infrared fluoride optical fibers, it is essential to take into account the heat accumulation on the fiber end face, particularly when increasing the pump power. While increasing the pump power, it can lead to potential damage to the fiber end face. Thus, it is often necessary to limit the pump power used to prevent any damage. In this particular case, the decision was made not to increase the pump power further to avoid any potential damage to the fiber end face. This approach guarantees that the optical fiber remains functional and reliable for its intended applications.

**Figure 8:**
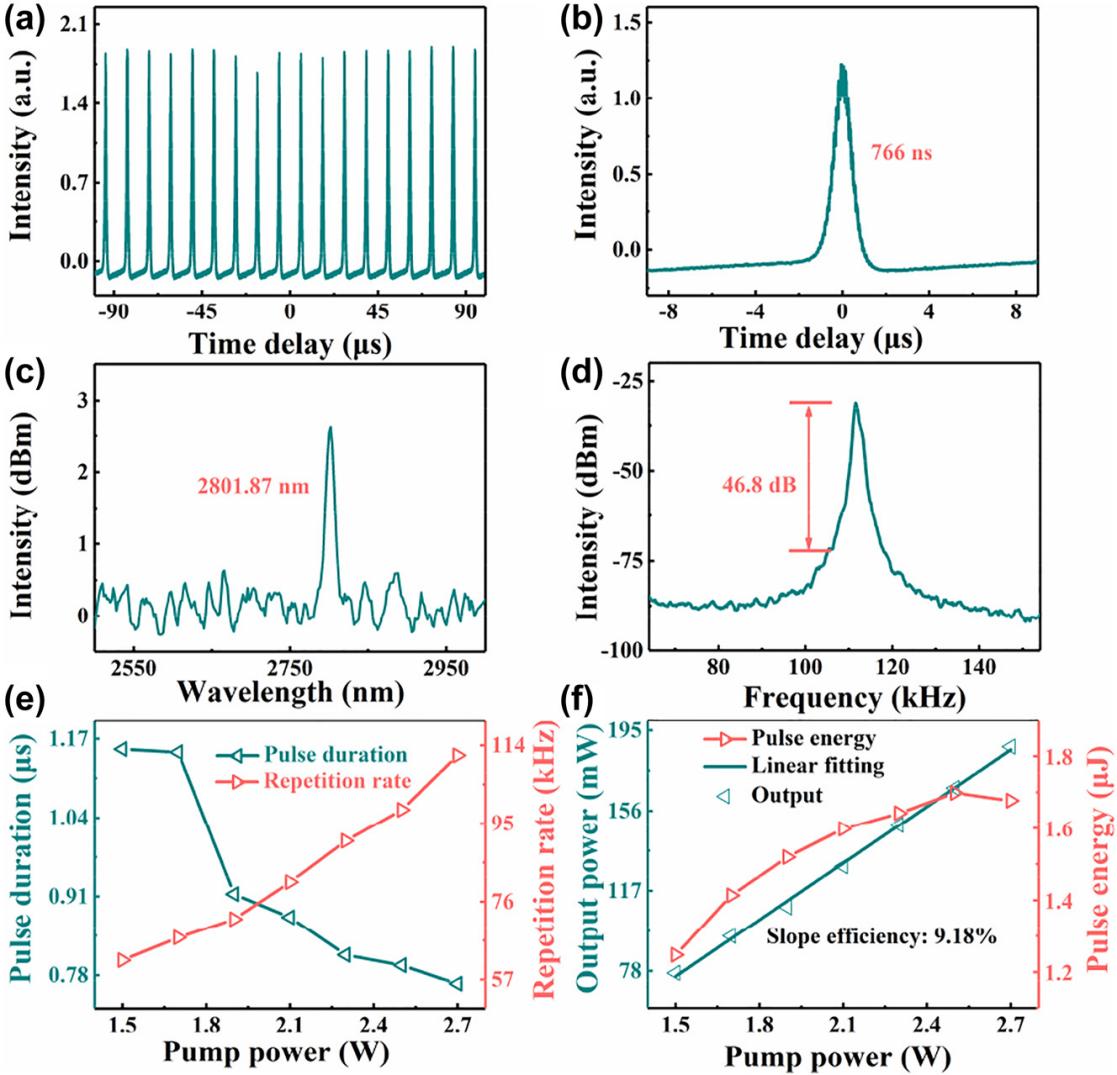
Characteristics of the output Q-switched pulse from EDZFL. (a) Pulse train observed on the oscilloscope at the pump power of 2.7 W. (b) Single pulse envelope measured by the oscilloscope. (c) Optical spectrum. (d) RF spectrum. (e) Pulse duration (blue triangle) and repetition rate (red triangle) change with pump power. (f) Output power (blue triangle) and pulse energy (red triangle) vary with input pump power.

Uniform Q-switched pulse trains and single pulse with a pulse width of 766 ns at the pump power of 2.7 W are depicted in Figure 8(a) and (b), respectively. Figure 8(c) depicts the output spectrum of the fiber laser whose center wavelength is 2801.87 nm. The RF spectrum of 46.8 dB illustrated in Figure 8(d) validates the stability of the Q-switched EDZFL. Figure 8(e) depicts the repetition rate and pulse duration as functions of pump power, and the Figure 8(f) depicts the evolution of pulse energy and output power in relation to the increasing of pump power. The output power increases linearly with the slope efficiency of 9.18 % with the increasing pump power, while the pulse energy increases from 1.25 μJ to 1.68 μJ accordingly. The results exhibit relatively low slope efficiency for the insertion loss of the SA and the non-optimized cavity length and the splicing loss, which can be improved by improving the transferring quality of the SA and optimize the fiber laser configurations in our future work. By applying the CrI_3_ film-SA to an EDZFL, the ability of CrI_3_ film as a nonlinear optical modulator has been validated for generating stable pulse lasers in the mid-infrared regime.

Besides, we have compared the output performance of the passively Q-switched and mode-locked fiber lasers based on the typical nonlinear optical materials in Tables 1 and 2, respectively. It is can be seen that from the tables that the fiber lasers modulated by the CrI_3_ film SA can deliver robust pulsed fiber lasers both in telecom wavelength and in the mid-infrared regime compared with other SAs. It is necessary to emphasize that the EDFL based on CrI_3_ film-SA in the optical communication band exhibits extraordinary stability with SNR of 92.4 dB, such a high SNR is due to the optimization of cavity parameters, including dispersion and the length of cavity, the stability of the pump source, the quality of the CrI_3_ film SA and so on, making it an excellent cost-effective solution required by high-speed and reliable data transmission applications.

**Table 1:** The performance summary of the Q-switched fiber laser based on different SAs in mid-infrared regime.

SA	Wavelength (nm)	∆*T* (%)	Repetition rate	Pulse width	SNR (dB)	Ref.
ITO NCs	2789	23.6	106 kHz	652 ns	43	[34]
Sb thin film	2789.5	7.1	201.5 kHz	824 ns	47.1	[35]
PtSe_2_	2783.2	10.2	93.1 kHz	1.04 μs	31.2	[36]
PbS	2710–3080	12.5	166.8 kHz	795 ns	33	[37]
CrI_3_ film	2801.87	18.07	111 kHz	766 ns	46.8	This work

**Table 2:** The performance summary of the mode-locked fiber laser based on different SAs in near-infrared regime.

SA	Wavelength (nm)	∆*T* (%)	Repetition rate	Pulse width	SNR (dB)	Ref.
Sb thin film	1562.64	11.77	–	753 fs	64.7	[35]
BP	1571.45	8.1	5.96 MHz	946 fs	70	[38]
VSe_2_	1565.8	19.11	12.8 MHz	714 fs	78.44	[39]
Ti_2_C_3_T_x_	1555.01	–	7.28 MHz	159 fs	62	[40]
GeAs_2_	1560	5.2	8.19 MHz	371 fs	60	[41]
GaSb	1559.1	2.1	15.28 MHz	585 fs	80	[42]
Bi_4_Br_4_	1559.23	42.3	49.92 MHz	172 fs	90	[43]
CrI_3_ film	1568.17	12.01	28.2 MHz	492 fs	92.4	This work

## 4 Conclusions

In summary, we have investigated the broadband nonlinear optical absorption behavior and applications of the CrI_3_ film in the telecom wavelength and the mid-infrared regime experimentally. The linear/nonlinear absorption and ultrafast response of the CrI_3_ film have been measured, demonstrating the ultrafast carrier dynamics and strong broadband saturation absorption towards the mid-infrared regime. With the excellent nonlinear optical absorption of the CrI_3_ film, robust pulsed fiber lasers based on CrI_3_ film-SA in 1.5 μm and 2.8 μm regions have been obtained experimentally. The mode-locked operation at 1567.17 nm showed extraordinary stability with a SNR of 92.4 dB and pulse duration of 492 fs. The stability of the mode-locked fiber laser has been confirmed through uninterrupted operation, indicating its reliability for highly stable optoelectronic applications. Meanwhile, a Q-switched EDZFL with a center wavelength of 2801.87 nm, SNR of 46.8 dB, and pulse duration of 766 ns has been demonstrated. The robust performance of CrI_3_ film as a broadband SA in telecom wavelength and mid-infrared regime highlights the potential for cost-effective optoelectronics devices, and may make inroads for developing highly stable broadband ultrafast optoelectronic devices.
